# Biomarkers and Psychological Factors Associated with Distress in Children, Adolescents, and Young Adults Undergoing MRI Neuroimaging: A Systematic Review of Observational Studies with Clinical Recommendations

**DOI:** 10.3390/healthcare14091160

**Published:** 2026-04-25

**Authors:** Guillermo Ceniza-Bordallo, Ana Belén del Pino, Dino Soldic, Angel Torrado-Carvajal

**Affiliations:** 1 Department of Psychiatry, Center for Health Outcomes and Interdisciplinary Research, Massachusetts General Hospital, Boston, MA 02134, USA; 2 Department of Psychiatry, Harvard Medical School, Boston, MA 02134, USA; 3 Research Group in Cognitive Neuroscience, Pain and Rehabilitation (NECODOR), Rey Juan Carlos University, 28032 Madrid, Spain; belen.delpino@urjc.es (A.B.d.P.); dino.soldic@urjc.es (D.S.); 4 Medical Image Analysis and Biometry Laboratory, Universidad Rey Juan Carlos, 28032 Madrid, Spain; angel.torrado@urjc.es

**Keywords:** chronic pain, distress, pediatric neuroimaging, magnetic resonance imaging, youth

## Abstract

**Introduction**: Distress during pediatric magnetic resonance imaging (MRI) neuroimaging can compromise scan quality and negatively impact children’s experiences. This review aimed to systematically synthesize biomarkers and psychological factors associated with distress in children, adolescents, and young adults undergoing neuroimaging. **Methods**: This systematic review was conducted according to PRISMA and AMSTAR-2 guidelines and preregistered in OSF. A systematic search was performed in six electronic databases, including observational articles published between 2000 and 2025 that assessed distress during MRI and functional MRI (fMRI). Data extraction and risk of bias assessment (QUIPS tool) were performed independently by two reviewers. **Results**: Ten studies (n = 558) examining distress during neuroimaging were included in this review. Distress was assessed through subjective self- and parent-reports, objective physiological measures, and qualitative interviews. Overall, distress levels were low to moderate; most participants tolerated scans well, though younger age, male sex, parental anxiety, procedure length, and chronic illness were associated with greater discomfort. Noise, immobility, and boredom emerged as the most frequent triggers, while strategies such as distraction, age-appropriate information, and reducing waiting times were perceived as helpful. Among participants with cancer, scan-related anxiety was closely linked to fear of recurrence and perceived stress. Risk of bias across studies was moderate to high, particularly in domains of attrition and statistical reporting. **Conclusions**: Distress during scanning is driven by anticipatory and parental anxiety, procedure length, and chronic illness. Biomarkers (e.g., cortisol, blood pressure) showed inconsistent links with subjective distress, highlighting the need for integrated measures.

## 1. Introduction

Magnetic resonance imaging (MRI) neuroimaging is an essential tool in both clinical and research settings for evaluating brain structure and function in children, adolescents, and young adults [1,2]. It is widely used for diagnosing neurological disorders, monitoring disease progression, and gaining insights into brain development [2,3]. However, despite its non-invasive nature, MRI procedures can be highly distressing for young patients [3,4].

Distress during MRI procedures is influenced by multiple factors, including patient-related characteristics, procedural aspects, and environmental conditions [4]. In relation to the procedure, the combination of a confined scanning environment, loud noises from the machine, extended periods of immobility, and an unfamiliar clinical setting can trigger significant anxiety, discomfort, and even physiological stress responses in children and youth [5,6,7]. In turn, these reactions can impact scan quality, leading to motion artifacts, failed imaging attempts, or the need for repeat scans, ultimately affecting both the efficiency and accuracy of neuroimaging studies [8,9]. Notably, cognitive mechanisms may also contribute to distress, as individuals can overestimate the likelihood or severity of negative experiences associated with medical procedures, thereby amplifying anxiety responses (e.g., probability overestimation and threat appraisal biases) [10]. For instance, catastrophic interpretations of scanner-related sensations (e.g., noise or confinement) and heightened attentional bias toward perceived threat cues may increase anticipatory anxiety, reinforcing expectations of discomfort prior to and during the procedure [11,12].

On the other hand, individual factors, such as age, temperament, trait anxiety, and previous medical experiences, can determine how a child responds to the imaging process [6,7,13]. For instance, younger children and those with heightened anxiety or prior negative medical encounters tend to exhibit higher distress [14]. Beyond self-reported and behavioral manifestations of distress, increasing attention has been directed toward the use of physiological biomarkers as objective indicators of stress responses during MRI procedures. Biomarkers such as heart rate and heart rate variability, skin conductance, and salivary cortisol have been used in pediatric and youth populations to capture autonomic and neuroendocrine stress responses during medical and experimental procedures, including neuroimaging contexts [15,16]. Previous studies have shown that elevations in physiological arousal during MRI are associated with heightened anxiety, reduced task engagement, and increased motion, underscoring the limitations of relying solely on subjective measures of distress [17]. Importantly, biomarker-based indicators may be particularly informative in younger children or highly anxious individuals, for whom self-report measures may be less reliable or feasible [18]. Incorporating physiological biomarkers alongside psychological and contextual factors is therefore critical for developing comprehensive models of distress and informing targeted strategies to improve scan tolerance and data quality [19].

Parental anxiety and attitudes toward the procedure also play a crucial role, as children often mirror their caregivers’ emotions [6,7]. Procedural factors, such as scan duration, noise levels, and physical discomfort associated with remaining still, further contribute to the overall experience [5,20]. Additionally, the presence or absence of preparation strategies, such as mock scanner training, audiovisual distractions, or cognitive-behavioral interventions, may significantly influence distress levels [21,22].

The consequences of distress extend beyond the emotional and psychological impact on the child. High distress levels can compromise the success of the imaging procedure by increasing motion artifacts, leading to the need for sedation or general anesthesia [21]. While sedation ensures compliance and reduces motion-related artifacts, it carries additional risks, including potential adverse reactions, increased healthcare costs, and logistical challenges for medical teams [23,24]. As a result, there is a growing interest in non-pharmacological approaches to managing distress, such as preparatory education, virtual reality exposure, relaxation techniques, and environmental modifications within imaging facilities [21].

Although distress during MRI has been most extensively studied in pediatric populations, emerging evidence suggests that adolescents, and young adults may experience comparable levels of anxiety and discomfort during scanning procedures [13,25]. This is particularly relevant given the ongoing maturation of cognitive and emotional regulation systems into early adulthood, as well as the continuity of procedural characteristics across age groups [26,27]. Including children, adolescents, and young adults allows for a developmental perspective on distress responses, acknowledging shared vulnerabilities while capturing age-related differences in coping, perception, and physiological stress reactivity.

Despite the recognition of distress as a major challenge in neuroimaging, the existing literature remains fragmented. Prior studies have predominantly examined distress from either a psychological or procedural perspective, often focusing on isolated outcomes such as self-reported anxiety or behavioral responses. In addition, existing reviews have largely centered on pediatric populations alone, with limited attention to adolescents, and young adults, and have rarely integrated objective physiological biomarkers alongside psychological and contextual factors. As a result, there is currently no comprehensive synthesis that simultaneously examines biomarkers, psychological processes, and clinical implications of MRI-related distress across developmental stages. This lack of integration limits the ability to develop mechanistically informed and clinically actionable strategies to reduce distress and optimize neuroimaging outcomes.

In this context, this systematic review aims to: (a) examine the biomarkers and psychological factors associated with distress during MRI neuroimaging procedures in children, adolescents, and young adults, (b) characterize the levels and type of distress experienced by children, adolescents, and young adults during neuroimaging procedures, (c) provide evidence-based strategies and recommendations to reduce distress and improve neuroimaging outcomes, with the goal of enhancing both patient experience and data quality.

## 2. Methods

This systematic review was conducted following the guidelines of the most recent guidelines Preferred Reporting Items for Systematic Reviews (PRISMA) [28], and A MeaSurement Tool to Assess systematic Reviews (AMSTAR 2) [29]. The review protocol was preregistered in Open Science Framework (OSF: osf.io/rs2bw). No deviations from the protocol were made.

### 2.1. Literature Search

Two independent reviewers (G.C.B & A.T.C) carried out a systematic search following the step-by-step guide described by Egger 2022 [30] for human studies. The search was performed in the following electronic databases, MEDLINE, PsycINFO, Embase, Web of Science, Cochrane Library, and CINAHL, covering literature published between 1 January 2000 and 1 January 2025, with no language restrictions. Detailed search strategies for each database are provided in Supplementary Material S1. The search strategy combined medical terms (MeSH) and keywords (“Distress Factors”, “Scan Process”, “Neuroimaging”, “Success Rate”), with limits applied for “humans” and “papers”. The search was last updated on 29 August 2025.

### 2.2. Eligibility Criteria

#### 2.2.1. Type of Studies

This systematic review included original, prospective observational studies, such as cross-sectional, case-series, cohort, and case–control studies, that analyzed at least one distress factor experienced by children, adolescents, and young adults during the MRI neuroimaging process. Eligible studies assessed at least one of the following: factors contributing to distress, levels of distress experienced by the child or their parents, the success rate of image acquisition, or the use of image correction techniques.

To ensure a focused analysis on non-pharmacological distress factors in pediatric neuroimaging, the following studies were excluded: (1) studies relying solely on sedation or general anesthesia as the primary strategy for distress management, as these do not provide insight into non-pharmacological approaches; (2) randomized and non-randomized clinical trials, case reports, conference abstracts, and review articles, because they do not provide systematically analyzable original data; (3) studies examining distress in medical procedures unrelated to neuroimaging, to ensure findings are specific to MRI neuroimaging in children and adolescents.

#### 2.2.2. Type of Participants

The population of interest consisted of children, adolescents, and young adults aged 4 to 25 years. To minimize confounding variables, this review focused on participants with typical neurodevelopment and excluded individuals with neurodevelopmental disorders, including autism spectrum disorder (ASD), attention-deficit/hyperactivity disorder (ADHD), and severe intellectual disabilities. Studies involving neuroimaging performed for emergency purposes, such as head trauma, stroke, or tumor evaluation, were also excluded, as these scenarios may introduce distress factors unrelated to standard neuroimaging procedures.

#### 2.2.3. Type of Neuroimaging Modalities

This review included studies examining distress associated with MRI and functional MRI (fMRI) neuroimaging.

#### 2.2.4. Outcome Measures

The primary outcomes of interest were distress levels and strategies to mitigate distress. Distress levels were measured using both objective and subjective indicators. Objective measures included physiological biomarkers such as heart rate variability, cortisol levels, respiratory rate, and blood pressure. Subjective measures comprised validated and non-validated self-reported questionnaires assessing fear of a confined scanning environment, loud machine noises, prolonged immobility, and/or an unfamiliar clinical setting, as well as parental assessments of distress. These measures allowed for a comprehensive evaluation of the anxiety and discomfort experienced by children during neuroimaging procedures.

#### 2.2.5. Factors Associated with Distress

This review included studies that analyzed factors associated with distress during neuroimaging procedures. Those factors were assessed prior to imaging and encompassed cognitive and psychological components, such as trait anxiety, psychiatric conditions, and fear of pain, which may predispose children to higher distress levels. Additionally, behavioral and attitudinal factors related to fear or avoidance of medical procedures were examined, as they can contribute to non-compliance during MRI neuroimaging. Sociodemographic variables, including age and sex, were considered for their potential influence on distress susceptibility. In addition, previous medical experiences, such as past neuroimaging procedures, frequent medical interventions (e.g., blood draws or vaccinations), and chronic medical conditions, were evaluated for their potential impact on the likelihood of distress.

### 2.3. Study Selection

Duplicate records were first identified and removed using MENDELEY^®^. The study selection process was conducted in two phases following the recommendations of A MeaSurement Tool to Assess systematic Reviews (AMSTAR 2) [29]. In the first phase, two blinded reviewers (G.C.B & D.S) assessed the eligibility of identified studies based on titles and abstracts. Any discrepancies were resolved by a third reviewer (A.T.C). In the second phase, the same reviewers (G.C.B & D.S) independently assessed the full-text articles for final inclusion. Disagreements regarding eligibility were resolved through discussion with the third reviewer (A.T.C) to reach a consensus. Inter-rater agreement during both phases was calculated using the kappa statistic, with scores interpreted as follows: *0.21–0.40* fair agreement, *0.41–0.60* moderate agreement, *0.61–0.80* substantial agreement, and *0.81–1.00* almost perfect agreement [31].

### 2.4. Data Extraction and Management

Data from the selected studies were independently extracted by two reviewers (D.S and A.B.P.M) into separate Excel^©^ spreadsheets, ensuring blind data collection. Extracted information included bibliographic details (first author, year of publication, and country), sample characteristics (sex and age) scanner type (e.g., MRI, fMRI), and scan characteristics (power, intensity, procedure duration, and machine configuration). Distress-related data were also extracted, including objective measures (e.g., heart rate, blood pressure) and subjective measures (e.g., discomfort, anxiety, fear), along with the questionaries or assessment tools used.

### 2.5. Methodological Quality Assessment

Risk of bias was assessed independently by two reviewers (G.C.B and A.T.C) using the Quality in Prognostic Studies (QUIPS) tool [32]. Initially, both reviewers scored two studies independently and discussed the results to standardize the application of the tool. Subsequently, all included studies were scored independently, with any disagreements resolved through consensus. Inter-rater agreement during this process was calculated using the kappa statistic, with interpretation criteria described below.

QUIPS [32] is a standardized instrument designed to evaluate risk of bias in prognostic factor studies. It assesses six key domains: (1) study participation, (2) study attrition, (3) prognostic factor measurement, (4) outcome measurement, (5) study confounding, and (6) statistical analysis and reporting. Each domain is rated as having low, moderate, or high risk of bias, providing a structured framework to ensure methodological rigor and transparency in systematic reviews of prognostic research.

## 3. Results

### 3.1. Studies Selection

**Figure 1:**
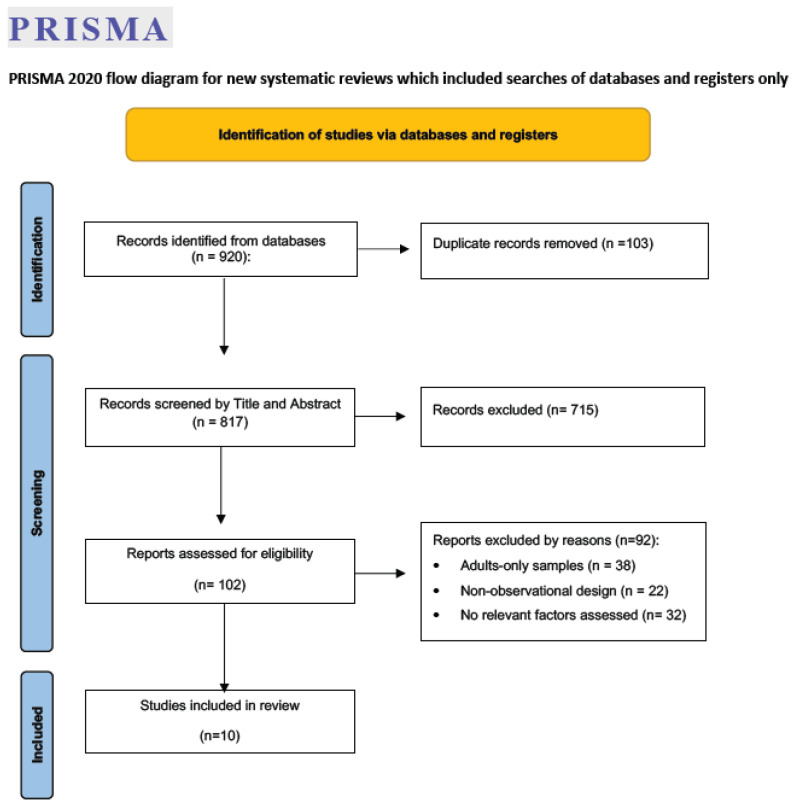
PRISMA 2020 flow diagram of study selection. Description: The flow diagram illustrates the process of study identification, screening, eligibility assessment, and inclusion.

The systematic search retrieved 817 articles after removing duplicates. Following title and abstract screening, 102 studies were selected for further evaluation. Of these, 92 were excluded for not meeting the eligibility criteria, resulting in a total of 10 studies [5,6,7,13,14,20,25,33,34,35] included in this review (see Figure 1). Discrepancies in eligibility assessment occurred in only one article and were resolved through discussion with a third reviewer. Inter-rater reliability between the two primary reviewers was excellent, with a kappa coefficient of 0.81, indicating almost perfect agreement [28].

**Figure 2:**
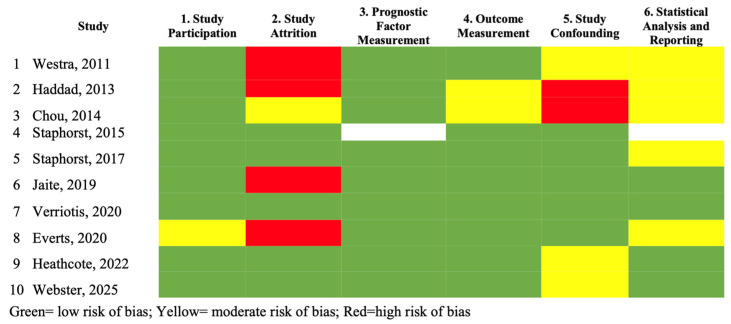
Risk of bias assessment of included studies. Description: Risk of bias was evaluated using the QUIPS tool across six domains: (1) study participation, (2) study attrition, (3) prognostic factor measurement, (4) outcome measurement, (5) study confounding, and (6) statistical analysis and reporting. Colors indicate the overall risk of bias for each domain: green = low risk, yellow = moderate risk, and red = high risk [5,6,7,13,14,20,25,33,34,35].

Across the 10 studies included in this review [5,6,7,13,14,20,25,33,34,35], the kappa index for agreement between the two evaluations was *0.79*, indicating good concordance [31] (see Figure 2). No studies showed a low risk of bias across all domains. The domains most prone to bias were “Study Attrition”, and “Statistical Analysis and Reporting”. It was not possible to evaluate the “Prognostic Factor Measurement” and “Statistical Analysis and Reporting” domains in Staphorst et al., (2015) [7] because it was a qualitative study. The rationale for each study’s scores is provided in Supplementary Material S2.

### 3.2. Participants Characteristics

Among the 10 studies included in this review [5,6,7,13,14,20,25,33,34,35] (n = 558 participants), 7 studies reported sex distribution [5,13,14,20,25,33,34], and participant ages ranged from 5 to 25 years. Considerable variability was observed in the clinical characteristics of study populations. Haddad et al. (2013) [14] included adolescents with clinical or subclinical anxiety. Verriotis et al. (2020) [34] focused on children with neuropathic pain, while Everts et al. (2020) [35] examined children born preterm and pediatric cancer survivors, comparing these groups with healthy controls. Another study [7] investigated children with inflammatory bowel disease, and Heathcote et al. (2022) [25] studied only childhood cancer survivors. The remaining four studies included healthy pediatric participants [5,6,20,33].

Overall, approximately half of the included studies focused on clinical populations, while the remainder examined healthy participants, reflecting a balanced but highly heterogeneous sample composition. This variability in underlying health conditions is likely to influence both baseline levels of distress and responses to MRI procedures, as clinical populations may present with heightened emotional burden or prior medical experiences, particularly in populations with chronic or severe conditions such as cancer or chronic pain.

### 3.3. Scan Characteristics

Eight of the included studies reported MRI procedures [5,6,7,13,20,25,33,34,35], one reported fMRI alone [14], and one included both MRI and EEG [33]. Both head and body scans were performed across studies. Specifically, Westra et al. (2011) [20] examined 19 participants undergoing head MRI and 35 undergoing body MRI. Scan durations varied widely, ranging from 10 to 90 min, with most studies reporting relatively short imaging times of approximately 30 min. For instance, Chou et al. (2014) [5] performed both head and musculoskeletal MRI in children with scan durations between 20 and 60 min, while Staphorst et al. (2017) [6] assessed distress during multiple medical procedures, including head and body MRI, in 89 children aged 10–17 years. In a separate analysis, Staphorst et al. (2015) [7] reported body MRI scans in children with inflammatory bowel disease, alongside other diagnostic procedures. Furthermore, Everts et al. (2020) [35] evaluated both healthy children and those born preterm, who underwent MRI, although scan durations were not reported. Similarly, Heathcote et al. (2022) [25] examined childhood cancer survivors undergoing a range of diagnostic procedures (e.g., X-ray, CT, electrocardiogram), including MRI. However, no specific information was pro-vided regarding MRI scan regions or durations, as was also the case in Webster et al. (2025) [13]. Regarding the combined imaging modalities, Verriotis et al. (2020) [34] performed head MRI and fMRI in children with neuropathic pain, reporting an average scan duration of 30 min. Haddad et al. (2013) [14] examined fMRI in children with clinical or subclinical anxiety, with an average scan duration of 60 min. Lastly, Jaite et al. (2019) [33] examined two pediatric groups (ages 8–17 years), one undergoing MRI (mean duration 11.3 min) and the other EEG (mean duration 11.1 min), with all procedures clinically indicated. An additional adult MRI group was also included in the original study but was excluded from this review.

Overall, substantial heterogeneity was observed in scan characteristics, including imaging modality, scan duration, and anatomical region, all of which are likely to influence the distress experience. Longer scan durations and procedures requiring extended immobility may increase discomfort and anxiety, particularly in younger participants, whereas shorter or less demanding protocols may be better tolerated. In addition, the inclusion of different imaging modalities (e.g., structural MRI, fMRI, EEG) introduces further variability in cognitive and sensory demands, potentially affecting distress responses across studies. The frequent lack of detailed reporting on scan parameters in several studies further limits the ability to systematically compare findings and identify procedure-specific contributors to distress.

### 3.4. Distress Assessed

The included studies assessed distress and suffering using a range of approaches. A range of distress-related but distinct constructs, including anxiety, fear, discomfort, and physiological stress responses, were measured both during scanning and retrospectively, and in some studies, participants were asked to compare their experiences with other potentially distressing medical procedures (see Table 1).

A small number of studies included exploratory physiological measures, such as cardiac variability, blood pressure, salivary cortisol, and physical activity, which were heterogeneous in methodology and not directly comparable. For instance, Westra et al. (2011) [20] examined cardiac variability and salivary cortisol after scanning, while Jaite et al. (2019) [33] assessed heart rate and blood pressure immediately before and after the imaging procedures. In contrast, Heathcote et al. (2020) [25] used physical activity as an objective indicator of physiological stress or autonomic arousal, asking participants to upload screenshots of their ambulatory step counts recorded via the Health App (iOS) or Google Fit App (Android) as part of an evening survey.

Subjective experiences were assessed using a variety of validated tools targeting anxiety, fear, discomfort, and broader affective states. Westra et al. (2011) [20] assessed children’s emotional states before and after MRI using the Face Image Scale (ranging from very happy to very unhappy, with a neutral midpoint) and asked participants to rate their overall experience on a four-point “How it was” scale (Pleasant-Unpleasant). Haddad et al. (2013) [14] evaluated state anxiety before, during, and after scanning with the State–Trait Anxiety Inventory for Children (STAI-C) and included ratings of perceived enjoyment. Staphorst et al. (2017) [6] combined measures of discomfort (CDRPQ), anxiety (STAI-C), and behavior (CBCL), while incorporating open-ended questions on strategies to reduce procedural discomfort. Chou et al. (2014) [5] assessed children’s perceptions of noise, cold, general discomfort, dizziness, claustrophobia, metallic taste, and twitch sensations using non-validated questions. Jaite et al. (2019) [33] measured state and trait anxiety, physiological hyperarousal, and subjective experience before and after MRI procedures. Verriotis et al. (2020) [34] assessed post-scan discomfort, perceived risk, and acceptability of current and future MRI procedures using a child-reported numeric rating scale (NRS). Everts et al. (2020) [35] evaluated trauma-related stress symptoms using the CRIES-8 scale, adapted from adult studies assessing “scanxiety”, and measured fear of cancer recurrence using the child version of the Fear of Cancer Recurrence Inventory (FCRI-C), along with body sensations during the procedure. Post-scan, ecological momentary assessment (EMA) was used to capture distinct but related domains including fear of cancer recurrence, perceived stress, affective states (e.g., anxiety, sadness, anger), and somatic symptoms. Staphorst et al. (2015) [7] conducted semi-structured interviews to explore children’s perceived discomfort during the procedure, preparation before the examination, willingness to undergo similar procedures in the future, and strategies to minimize procedural burden and negative affect. These interviews also addressed specific symptoms such as pain, nausea, breathing difficulties, anxiety, fatigue, and boredom, among others.

Parental and staff assessments were also incorporated in several studies. Westra et al. (2011) [20] evaluated children’s anxiety (before and after MRI) and cooperation (after the procedure) based on reports from both parents and MRI staff, while parents’ trait anxiety was assessed prior to the scan using the Spielberger State–Trait Anxiety Inventory. Chou et al. (2014) [5] collected parents’ perceptions of the procedure’s tolerability for their children. Finally, Verriotis et al. (2020) [34] evaluated parental perceptions of post-scan discomfort (affective/behavioral response), perceived risk, and acceptability of current and future MRI procedures using an NRS.

Overall, the assessment of distress was highly heterogeneous across studies, encompassing subjective self-reports, physiological biomarkers, and proxy reports from parents and healthcare professionals. While subjective measures were the most frequently used, they varied considerably in terms of constructs assessed, timing (pre-, during, or post-scan), and use of validated versus non-validated tools, limiting direct comparability across studies. In contrast, physiological measures were less commonly implemented and showed substantial variability in methodology, precluding consistent interpretation of objective stress responses. Notably, discrepancies may arise between subjective and physiological indicators of distress, as subtle autonomic responses may not always be reflected in self-reported experiences. The inclusion of parental and staff reports adds a valuable multi-informant perspective but may also introduce additional variability due to differences in perception and reporting. Taken together, the lack of standardized and harmonized approaches to assessing distress represents a major challenge for synthesizing findings and identifying consistent patterns across studies.

### 3.5. Distress Outcomes Reported by Participants

Across studies, distress-related outcomes during pediatric neuroimaging were generally rated as low to moderate, though several specific triggers and influencing factors were identified (see Table 2). Only a minority of children reported negative affect, perceived discomfort, anxiety, or physiological arousal during MRI [33], and many described the experience as neutral or even positive. For instance, 40% of participants reported feeling happy after the scan [20]. Haddad et al. (2013) [14] observed no significant differences in anxiety levels between anxious and non-anxious groups either before the scan or at the onset of scanning. However, significant group differences emerged immediately after the scan (*p* = *0.001*) and one-hour post-scan (*p* = *0.002*), though both groups had returned to baseline levels by the time the participants arrived home. Interestingly, children with higher trait anxiety reported greater enjoyment, and trait anxiety scores were positively correlated with willingness to undergo future scans [14]. The most commonly reported sources of sensory and situational discomfort included scanner noise, cold, immobility, and boredom, particularly during procedures lasting longer than 60 min or when children were undergoing MRI for the first time [5,7]. Claustrophobia, dizziness, and physical sensations such as metallic taste or twitching were reported less frequently [5]. Regarding physiological measures, patterns were mixed, with salivary cortisol levels increasing during scans but not consistently associated with self-reported discomfort [20], and the use of contrast agents was linked to higher cortisol levels [20]. Heart rate and systolic blood pressure remained largely stable, although diastolic blood pressure increased after MRI, particularly among first-time patients [33]. Parental anxiety also influenced outcomes, with higher parental state anxiety associated with greater perceived child anxiety and lower reported cardiac variability in children [20], while younger children were more often perceived as anxious. While most parents rated the scans as tolerable, they frequently noted discomfort related to prolonged immobility or the hardness of the mattress [5]. Age and sex differences were observed in preterm birth groups, where younger children reported higher discomfort and boys reported more discomfort than girls, although no sex differences emerged for fear [35]. In the healthy control group, neither discomfort nor fear varied by sex, and fear related to MRI procedures decreased significantly from childhood to adolescence among patients, whereas discomfort remained stable across ages [35]. Psychological context and chronic illness strongly influenced scan-related distress. Among children with cancer or chronic pain, scanxiety was closely associated with fear of cancer recurrence and perceived stress [13,25]. Anticipatory bracing and preparation predicted sharper increases in fear of cancer recurrence in the days before scanning, whereas positive coping strategies such as optimism or hope were not protective [13]. In children with neuropathic pain, discomfort and acceptability ratings were generally favorable, though adolescents expressed more worry and lower willingness to undergo future scans than parents [34]. Children also highlighted strategies to reduce distress, including minimizing waiting times and administrative burdens, providing distractions during scans (e.g., movies, music), avoiding redundant procedures in those with chronic illness, and delivering age-appropriate information. Younger children particularly valued receiving small rewards such as stickers or diplomas to acknowledge their participation [6,7]. Overall, most children tolerated neuroimaging procedures well, although this was context-dependent, with anticipatory anxiety, procedure length, parental anxiety, and clinical context emerging as key drivers of reported outcomes, including anxiety, fear, discomfort, and physiological stress responses.

Taken together, despite the overall low to moderate levels of reported distress, a consistent pattern emerges whereby distress appears to be driven by three main domains: (1) procedural factors (e.g., scan duration, noise, immobility), (2) individual characteristics (e.g., age, trait anxiety, prior experiences), and (3) contextual influences (e.g., parental anxiety, clinical condition). Notably, discrepancies between subjective reports and physiological indicators suggest that distress may not be fully captured by self-report measures alone. In addition, clinical populations, particularly those with chronic illness, appear to experience distress that is more closely related to the broader emotional context of the disease (e.g., fear of recurrence) rather than the technical aspects of the MRI procedure itself. This structured interpretation helps to explain the variability observed across studies and highlights the multifactorial and context-dependent nature of MRI-related distress.

## 4. Discussion

The purpose of this systematic review was to examine the scientific literature on factors associated with distress during neuroimaging procedures in children, adolescents, and young adults. By collecting and critically evaluating prospective observational studies, this review highlights that distress, a term encompassing anxiety, fear, discomfort, and physiological stress responses, is a multifactorial phenomenon influenced by individual characteristics, procedural aspects, parental anxiety, and clinical context. Overall, most children and young adults tolerated neuroimaging procedures well, yet anticipatory anxiety, procedure length, noise, immobility, and chronic illness emerged as key contributors to discomfort. Objective and subjective assessments, including physiological measures, self-reports, and parental observations, revealed that distress is generally low to moderate. However, results are heterogeneous and context-dependent, with certain subgroups, such as younger children, first-time patients, and those with chronic conditions, appearing more vulnerable. The lack of standardized distress measures across studies should also be considered when interpreting these findings, as variability in measurement approaches may partly account for inconsistencies in reported outcomes and limit the strength of the overall conclusions. Furthermore, the relatively small number of included studies (n = 10) suggests that the current evidence base is still emerging, which should be considered when interpreting the robustness and generalizability of the findings.

A relevant aspect of the present review is the inclusion of both healthy and clinical populations, which may further contribute to variability in distress-related outcomes. While some studies were conducted exclusively with healthy children and adolescents, others focused on clinical samples with specific diagnoses, such as anxiety disorders or cancer. This diversity introduces an additional level of complexity to the interpretation of findings, as the presence of an underlying medical condition may substantially modulate the emotional and physiological experience associated with MRI procedures.

In the case of patients with anxiety disorders, it is possible that distress levels during MRI may be amplified by a generalized hypersensitivity to novel or perceived threatening situations, such as enforced immobility, scanner noise, or the lack of control over the procedure. Accordingly, children with high trait anxiety would be expected to exhibit greater vulnerability to MRI-discomfort. However, a study conducted by Shechner and colleagues (2013) [36] shows that children with anxiety did not report higher levels of distress after undergoing an fMRI procedure compared with healthy children, suggesting that the technique itself does not necessarily exacerbate anxious symptoms. This finding raises the possibility that even among patients diagnosed with anxiety, the fMRI experience may be perceived as tolerable and associated with relatively low levels of psychological distress, thereby supporting the feasibility of its widespread use in this population.

On the other hand, in patients with illnesses such as cancer, distress during MRI may be influenced not only by technical aspects of the procedure but also by the emotional burden associated with the underlying disease and its potential progression. The literature on adult cancer populations reinforces this interpretation, as it has been reported that MRI-related emotional burden extends beyond the scanning moment, encompassing the period prior to the procedure, the anticipation of results, the act of reviewing them, and even the reception of an unfavorable diagnosis [37]. In this context, adults have expressed the need for more information, communication, and empathy from healthcare professionals, in addition to reporting the use of multiple self-coping strategies to manage distress before, during, and after the scan [37]. Although these data derive from adult populations, they support the hypothesis that children with cancer may benefit from interventions that integrate not only technical preparation for the procedure but also sustained psycho-emotional support throughout the diagnostic process.

Importantly, the moderate to high risk of bias identified across studies, particularly in relation to attrition and statistical reporting, further limits the strength of these conclusions. These biases are not unexpected in MRI research involving pediatric and young populations, where participation requires sustained immobility, tolerance to a confined and noisy environment, and, in some cases, repeated assessments. Children or adolescents with higher levels of anxiety, lower distress tolerance, or greater sensitivity to sensory stimuli may be more likely to withdraw or be excluded due to excessive motion, potentially introducing selection bias and leading to an underestimation of distress levels. In addition, variability in statistical reporting and analytical approaches across studies may reflect the lack of standardized protocols in this field, further limiting comparability and synthesis of findings.

Significantly, one of the most consistent findings of this review is the lack of concordance between physiological biomarkers and subjective reports of distress. This discrepancy suggests that physiological activation does not necessarily translate into consciously perceived anxiety or discomfort. In other words, individuals may exhibit elevated stress biomarkers (e.g., cortisol or autonomic responses) without reporting increased subjective distress. This pattern highlights that the experience of distress is not solely determined by physiological arousal, but is strongly shaped by subjective appraisal processes, including cognitive interpretation, expectations, and perceived control. As a result, subjective and objective measures may capture distinct, albeit complementary, components of the stress response. While methodological factors—such as differences in measurement timing, type of biomarkers, and population characteristics—may contribute to variability across studies, the observed dissociation appears to reflect a more fundamental distinction between physiological state and lived experience. These findings underscore the importance of integrating both subjective and physiological assessments to obtain a more comprehensive understanding of distress during neuroimaging procedures.

Taken together, differences observed across clinical samples make it difficult to reach uniform conclusions and limit the generalizability of findings to the pediatric population as a whole. Nevertheless, the available evidence suggests that, despite this heterogeneity, the impact of MRI-related distress appears to be limited in many children, although findings are heterogeneous and context-dependent. In particular, distress may be higher in conditions where the underlying diagnosis and its associated emotional burden constitute the main source of anxiety, rather than the technical procedure itself.

Essentially, the present review advances the current literature by providing an integrative and developmentally informed synthesis of MRI-related distress. While previous studies have often examined distress through isolated domains—such as self-reported anxiety, behavioral responses, or procedural factors—this review brings together evidence across psychological, physiological, and contextual dimensions, including the role of objective biomarkers. In addition, by extending the scope beyond pediatric populations to include adolescents, and young adults, this work offers a broader developmental perspective on distress responses during neuroimaging. This integrative approach contributes to a more comprehensive understanding of the mechanisms underlying distress and supports the development of more targeted and clinically relevant strategies to improve patient experience and data quality

### 4.1. Recommendations to Reduce Distress During Scanning Procedures in Children, Adolescents, and Young Adults

The following recommendations are informed primarily by findings from the included studies, particularly regarding preparation strategies, communication approaches, and environmental adaptations. Where relevant, these recommendations are further contextualized and supported by broader literature to enhance their clinical applicability.

The available evidence suggests that the implementation of interventions aimed at reducing distress in pediatric patients undergoing MRI not only improves the patient’s overall experience but also significantly contributes to the acquisition of higher quality images, reducing motion related artifacts and the need for repeated scans. Among the most widely recognized strategies is prior familiarization with the scanner environment, which may include visits to the MRI or the use of mock scanners [36]. Such interventions allow patients to face the procedure in a controlled and gradual context, thereby reducing anticipatory anxiety and enhancing cooperation during the actual scan [3,36,38,39]

In addition to scanner familiarization, providing clear, age-appropriate information about the procedure constitutes an essential component in decreasing uncertainty and distress associated with the MRI experience [3]. Comprehensible explanations regarding scanner sounds, test duration, and the role of healthcare staff contribute to a greater sense of perceived control in both children and their parents, which in turn has been linked to lower levels of distress [36]. Moreover, guidance about the care team accompanying the patient and the role of parents is particularly relevant, since perceived social support has been identified as a key modulatory factor in the emotional response to hospital procedures [40].

Regarding specific interventions, various relaxation techniques, such as progressive muscle relaxation or age-appropriate relaxation imagery, have demonstrated benefits in reducing stress levels among older children and adolescents [41,42]. Complementarily, the integration of playful or distraction-based elements, such as audiovisual stimuli, music, or interactive games during the waiting period, represents an increasingly evidence based strategy in pediatric settings, as it facilitates emotional regulation and promotes adherence to the procedure [43,44]. This psychological and environmental preparation of patients emerges as a valuable tool, as it optimizes neuroimaging quality by minimizing motion artifacts while simultaneously improving the patient’s subjective experience, fostering willingness to participate in research studies, and enhancing overall procedural acceptability. The systematic implementation of such measures should be considered a priority recommendation in both clinical practice and research involving children and adolescents in neuroimaging studies.

### 4.2. Limitations

First, the number of included studies was relatively small (n = 10), which restricts the generalizability of the findings and limits the ability to identify consistent patterns regarding factors associated with MRI-related distress in pediatric, adolescent and young adults. Furthermore, the scope of this review is constrained by this limited evidence base, meaning that the robustness of the conclusions depends heavily on the available data.

Second, methodological heterogeneity represents a well-recognized challenge in this type of review. The included studies differed in key aspects such as participant inclusion criteria (e.g., underlying medical conditions and age ranges) and distress measurement tools (e.g., validated scales versus self-reports). This highlights the difficulty of comparing and synthesizing heterogeneous findings within the review process. Moreover, this heterogeneity suggests that distress should be interpreted as a context-dependent construct, varying according to factors such as anticipatory anxiety, parental influences, and underlying clinical conditions.

Third, the presence of bias cannot be ruled out. The reliance on self-reported measures introduces the risk of social desirability bias, particularly in pediatric populations, where additional factors such as comprehension of questionnaire items and developmental differences in the ability to self-report distress may vary with age and cognitive maturity.

An additional limitation relates to the lack of standardization in distress assessment across studies. A wide range of instruments was used, including both validated and non-validated measures, targeting partially overlapping constructs such as anxiety, fear, discomfort, and physiological stress. This variability complicates direct comparisons across studies and limits the ability to derive consistent estimates of distress levels. Moreover, the use of non-validated or study-specific measures may introduce measurement bias and reduce the reliability and interpretability of findings. As a result, the overall strength of the evidence is constrained, and conclusions regarding the magnitude and nature of MRI-related distress should be interpreted with caution.

Although this review was designed to focus on distress associated with MRI neuroimaging procedures, some of the included studies—particularly those involving clinical populations such as cancer survivors—reflect a broader conceptualization of distress that extends beyond the technical aspects of the scan itself. In these contexts, distress may be influenced not only by procedural factors, but also by the clinical meaning of the imaging, including disease monitoring, anticipation of results, and fear of recurrence. This highlights that MRI-related distress cannot always be disentangled from the wider diagnostic or clinical context, particularly in populations with chronic or severe conditions.

Finally, although the GRADE framework was applied to assess the quality of evidence, the limited number of studies and small sample sizes for some outcomes contribute to imprecision, resulting in low to very low certainty ratings, further limiting the generalizability and certainty of the findings.

## 5. Conclusions

These findings highlight the importance of targeted strategies to reduce distress, including preparation interventions, age-appropriate information, environmental modifications, and distraction techniques, as they may enhance both patient experience and imaging success. Nevertheless, these conclusions should be interpreted cautiously given the limited number of studies, risk of bias and their methodological heterogeneity.

## Figures and Tables

**Table 1:** Characteristics of included studies in this review.

Author, Year	Sample (% Female)	Age Range (Mean Age, Years)	Imaging Modality	Scan Duration	Distress Factors Analyzed and Scales Used
1. Westra, 2011 [20]	54 (61%)	5–12 (9.15)	MRI (Head n = 19 other n = 35)	20 min (10–90 min)	**Children:** Experienced feelings (Facial Image Scale) at two time points (before and after MRI) “How it was” (Pleasant-Unpleasant, Four-point scale) once (after MRI) Heart rate variability at four timepoints (before, 1, 5, and 10 min) Salivary cortisol at four time points (before and, immediately, one hour and the next day after the MRI) Comparison with other tests blood draw/vaccination, once (after MRI) **Parents and Staff:** Parents and MRI staff reported the child’s level of anxiety (five-point scale) at two time points (before and after MRI) Degree of cooperation of the child asked by the staff once (after MRI) Trait anxiety: (Spielberger State–Trait Anxiety Inventory) once (before)
2. Haddad, 2013 [14]	36 with clinical and subclinical anxiety (72%)	12–18 (15.72)	fMRI (head)	60 min	Trait Anxiety: STAI-C Self-reported Confidential Online Questionnaire (question 1–7: nine-point scale, question 8–9: five-point scale) Self-reported free text answers about best/worst aspects of study
3. Chou, 2014 [5]	31 underwent MRI for brain or musculoskeletal scans (45%)	7–17 (12.7)	MRI (3T); Sequences: T1; T2; FLAIR (head and musculoskeletal)	20–60 min	**Children:** Noise ^ Cold ^ General discomfort ^ Dizziness ^ Claustrophobia ^ Perception of metallic taste or sensation of twitches (only 8–17-year-olds) ^ Peripheral nerve stimulation (over 8 years old) Whether they would undergo another MRI Whether undergoing tests or receiving contrast agents would affect their decision (ages 12 to 17 years) **Parents:** Questionnaire assessing parents’ opinions on their children’s tolerability of 3T MRI ^
4. Staphorst, 2015 [7]	8 Inflammatory bowel disease (no data)	6–18 (no data)	MRI (body)	NR	Semi-structured interview: ○ Discomfort during procedure ○ Previous preparation ○ Willingness to repeat the experience ○ How to reduce discomfort Physical discomfort: pain, nausea, difficulty breathing, itching, hunger Psychological discomfort: anxiety, fatigue, boredom, embarrassment
5. Staphorst, 2017 [6]	89 MRI group (no data)	8–17 (10.5)	MRI (head and body)	30–60 min	General discomfort: CDRPQ Trait Anxiety: STAI-C Open-ended question on suggestions to reduce discomfort
6. Jaite, 2019 [33]	MRI (children group who underwent brain MRI scanning [MRI-C group]): 25 (56.1%) EEG (random children group who underwent EEG recording [EEG-C group]): 34 (48.5%)	8–17 (12.9) MRI: 12.7 EEG: 12.3	MRI (1.5T) and EEG (head)	MRI: 11.3 min (SD = 2.2) EEG: 11.1 min (SD = 2.0)	Anxiety: CBCL/4–18 State–Trait Anxiety: STAIC Physiological hyperarousal: PH-C Experience during the MRI: PEQ Heart rate: Sanitas SBM 03 blood pressure meter Blood pressure: Sanitas SBM 03 blood pressure meter
7. Verriotis, 2020 [34]	21 (52.43%) with neuropathic pain 18 (56%) filled post scan questionnaires	10–18 (14.6)	MRI (3T) with sequences (T1 and diffusion weighted images and resting-state fMRI) (head)	30 min	Pain intensity and activity interference due to pain: VAS Emotional distress: PI-ED Quality of life: PedsQL Pain catastrophizing: PCS-C **Both parents and children:** Distress during the MRI: NRS Risk perception: NRS Acceptability of current and future MRIs: NRS
8. Everts, 2020 [35]	212 (NR) 102 healthy control and 110 patients (born very preterm and childhood cancer)	7–18 (NR)	MRI (head)	NR	Fear/Discomfort: (Faces Scale 0–4): 0 = no discomfort/no fear, 1 = almost no discomfort/almost no fear, 2 = a little discomfort/a little fear, 3 = a lot of discomfort/a lot of fear, 4 = extreme discomfort/extreme fear; ranging from a friendly smiling face (0) to a very worried face (4) Fear and discomfort assessed on a separate scale Parental Educational Level (Swiss Education System): 1 = no graduation 2 = college 3 = college of higher education 4 = university degree Intelligence (WISC-IV and TONI-4) Inhibition (Color word interference task)
9. Heathcote, 2022 [25]	30 (47%) survivors of childhood cancer	11–25 (17.6)	X-ray, CT, MRI, echocardiogram, and laboratory tests	NR	**Baseline:** Scanxiety: CRIES-8 Fear of Cancer Recurrence: FCRI-C Bodily Threat: BTMS Ecological Momentary Assessment (EMA) surveys three times per day for 11 days around their routine surveillance scans: ○ Fear of cancer recurrence (two items of FCRI-C) ○ Body threat monitoring (BTMS) ○ Stress (Perceived Stress Scale) ○ Negative and Positive Affect (items adapted from the PANAS) ○ Self-checking behaviors (yes/no) ○ Somatic symptoms (14 items) ○ Social connectedness (two items) **At the end:** Feedback survey
10. Webster, 2025 [13]	50 (40%)	11–25 (17.13)	X-ray, CT, MRI, echocardiogram, and laboratory tests	NR	Uncertainty management: Participants completed four items that were adapted from other studies of stressful waiting orienting them to report how they manage uncertainty around surveillance scans ○ People sometimes adopt strategies for dealing with stressful life events. Please indicate how true each statement was for you leading up to your most recent scan or cancer check-up. ○ I was bracing myself for the worst possible outcome” and “I tried to keep expectations low for the outcome ○ I was hoping for the best possible outcome” and “I tried to be optimistic about the outcome All items were rated on a 7-point Likert scale (1 = strongly disagree; 7 = strongly agree). Fear of pain recurrency: FCRI-C

^ = not validated scales used for those variables (questionaries can be found at [5]); MRI = Magnetic Resonance Imaging; fMRI = Functional Magnetic Resonance Imaging; T = Tesla; EEG = Electroencephalogram; NR = Not reported; PANAS = Positive and Negative Affect Schedule; FCRI-C = FCR Inventory-Child Version; BTMS = Bodily Threat Monitoring Scale; CRIES-8 = Children’s Revised Impact of Events Scale; WISC-IV = Wechsler Intelligence Scale for Children; TONI-4 = Test of Nonverbal Intelligence; VAS = visual analogue scale; PI-ED = Pediatric Index of Emotional Distress; PedsQL = Paediatric Quality of Life Inventory; PCS-C = Pain Catastrophizing Scale-Children; NRS = Numerical Rating Scale; CBCL = Child Behaviour Checklist; CBCL/4-18 = parent-reported Child Behaviour Checklist 4-18; STAI-C = State–Trait Anxiety Inventory for Children; PEQ = Patient Experience Questionnaire; CDRPQ = Children’s Discomfort during Research Procedures Questionnaire.

**Table 2:** Main results from the included studies in this review.

Author, Year	Main Results Reported by Children	Main Results Reported by Parents
1. Westra, 2011 [20]	Before scan: 39/54 happy or very happy 4/54 sad or very sad During scan: 22/54 happy or very happy 10/54 sad or very sad After scan: 30/54 very pleasant or fairly pleasant 24/54 unpleasant or very unpleasant Physiological Measures: No increase in heart rate (median: −0.17 SDS, range: −1.29–1.53) Salivary cortisol increased (median: 0.71 nmol range: −8.96–36.16). Median increment of 23%. Subjective Experience: 40/54 children remembered undergoing a venipuncture or vaccination 4/40 children reported that the MRI was more unpleasant than the needle puncture	Parents with high state anxiety scores rate their child as more anxious (β = 0.373, *p* = 0.005), but with a lower SDS and lower heart rate (β = −0.395, *p* = 0.038).
2. Haddad, 2013 [14]	Before the examination: No significant difference in self-reported trait anxiety between the two groups was observed (*p* = 0.09). During the test: No significant difference in self-reported anxiety at the start (*p* = 0.15). Statistically significant difference by the end of the scan (*p* < 0.01) At the end of the test: A statistically significant difference emerged (*p* > 0.01) and remained significant one hour later (*p* = 0.02). Once at home: The group differences returned to the initial level (*p* = 0.06). Enjoyability of the MRI experience: No significant difference between anxious and non-anxious groups *t*-test, but there was trend-level correlation (*p* = 0.07). Those with higher trait anxiety reported greater enjoyment. Significant positive correlation between trait anxiety score and likelihood of having another scan (*p* < 0.01) No further statistically significant differences were found.	NR
3. Chou, 2014 [5]	Adverse Sensations: Noise (39%; 12/31) Cold (19%; 6/31) General discomfort (16%; 5/31) Dizziness (13%; 4/31) Claustrophobia (10%; 3/31) Adverse Effects: 1/31 (3%) reported a very uncomfortable experience 1/26 (4%) reported a metallic taste in the saliva 6/26 (23%) reported sensations consistent with possible peripheral nerve Stimulation (unusual movements and contractions) 22/28 (79%) Unwilling to repeat MRI within one year 8/19 (42%) children aged 12 to 17 years agree that having a blood test or having a contrast agent injection would put young people off volunteering for MRI. Only noisiness was significant between 7–11- and 12–17-year-old groups (*p* = 0.021) Quality of brain images: Images rated as blurry (2/29; 7%) were not related to patients reporting discomfort (*p* = 1.00)	**Parents:** Discomfort of their children caused by mattress hardness and time lying down (27%; 7/26). Other adults who had previously undergone 3 and 7 T MRI: They thought that children between 12 and 17 years of age would tolerate MRI well (85%). This percentage decreased when they thought of younger children (35% in ages 10 to 11 years; 15% in ages 8 to 9 years).
4. Staphorst, 2015 [7]	Qualitative findings: Initial anxiety: Most children only felt anxious during their first procedure. *“For me, [MRI] is quite normal once you know how it works. The first time I was a little scared, but now I’m used to it”–15-year-old boy.* Some of the healthy children and children with a mild chronic condition were somewhat anxious because they did not know what to expect from the procedures, the hospital environment and researchers: “The first time I was a little scared, because I did not know the people [i.e., researchers], now [that I know them] it is more pleasant” Boredom: Boredom was mentioned due to the lengthy procedures (lying in an MRI scanner for 60 min without distractions). Reasons for not wanting to participate again: Boredom (n = 3) Travel time (n = 3) Pain (n = 4) Poor scheduling of research visits that conflicted with regular medical appointments (n = 4) Recommendations to reduce discomfort: Reduce waiting times and administrative procedures. Provide distractions during uncomfortable and monotonous procedures (e.g., watching movies, listening to music, access to playrooms). Adapt playrooms for older children too. Children with chronic conditions suggested that tests conducted during the research should be used as part of their clinical care to avoid repetition. They also recommended that the study should not take place during school hours, whereas healthy children did not mind a school-time schedule. Information provision: Several children mentioned that the written information provided about the study should be more age-appropriate, as it was often too difficult to understand or lacked details about aspects that interested them (e.g., whether the MRI is painful, what the pre-test liquid tastes like). They believed that receiving more information would make them less anxious. Incentives: Some children indicated that they would have appreciated a small gift as a token of thanks (e.g., a movie gift card) for participating in the research study. Younger children specifically mentioned that they would like to receive a sticker or a diploma to feel proud of having undergone a research procedure, especially if it was painful. 2/8 children who underwent MRI had previous experience with this technique	NR
5. Staphorst, 2017 [6]	Nervous: 39%: not nervous 45%: slightly nervous 11%: a little nervous 2.5%: very nervous 2.5%: extremely nervous Annoyed/Irritated: 70%: not at all 18%: slightly 11%: a little 1%: very 0%: extremely Pain: 90%: no pain 10%: slightly 0%: a little 0%: very 0%: extremely Frightened: 70%: not scared 25%: slightly scared 5%: a little scared 0%: very scared 0%: extremely scared Bored: 48%: not bored 38%: slightly bored 9%: a little bored 1%: very bored 4%: extremely bored Tired: 30%: not tired 35%: slightly tired 12%: a little tired 8%: very tired 10%: extremely tired Suggestions to reduce discomfort: Less noise (24/89) Warner room temperature (3/89)	NR
6. Jaite, 2019 [33]	Group comparisons (MRI Children vs. MRI adults): No statistically significant differences were observed between children and adults, either before or after the MRI, regarding anxiety (*p* = 0.262 and *p* = 0.374, respectively) or physiological arousal (*p* = 0.050 and *p* = 0.472). Heart rate not decreased significantly during the procedure in the child group (*p* = 0.058). Systolic blood pressure did not change significantly in any group (*p* = 0.630 and *p* = 0.610). Diastolic blood pressure was significantly higher after the procedure in the child group (*p* = 0.044), but not in the adult group (*p* = 0.154). Child MRI vs. Child EEG Group: Situational anxiety: the abnormal values before and after the procedure were 21.8% and 12.7% in the child MRI group and 6.6% and 6.3% in the EEG group. Both groups did not differ significantly, either before or after their respective procedures, regarding anxiety (*p* = 0.525 and *p* = 0.875, respectively), physiological arousal (*p* = 0.53 and *p* = 0.189), systolic blood pressure (*p* = 0.561 and *p* = 0.190), or diastolic blood pressure (*p* = 0.257 and *p* = 0.773). There was no difference in heart rate before the procedure (*p* = 0.334), patients in the child MRI group had a significantly higher heart rate after the exam compared to those in the child EEG group (*p* = 0.006). First-Time vs. Experienced MRI: Children undergoing their first MRI scored significantly higher in physiological arousal after the examination (PH-C: *p* = 0.029) than those with previous MRI experience. No further significant differences between these groups were found in any other subjective or physiological variables. Predictors in the Child MRI Group: In the child MRI group, age, sex, IQ, participants’ trait anxiety scores, number of previous MRIs, and anxiety regarding MRI findings were not significant predictors of situational anxiety (STAI(C)-S; adjusted R2 = 0.058, F(6, 43) = 1.50, *p* = 0.201) or physiological arousal (PH-C; adjusted R2 = 0.123, F(6, 43) = 2.14, *p* = 0.068). Patient Experience: According to the patient experience questionnaire, over 98% of children reported feeling little or no anxiety during the MRI examination. The anxiety experienced by children undergoing an MRI did not differ from that experienced by those undergoing an EEG recording.	NR
7. Verriotis, 2020 [34]	Post scan acceptability and discomfort Ratings for current research scan acceptability were high for both adolescents (range [median]: 8–10 [10]; 67% rated 10/10) and parents (7–10 [10]; 81% rated 10/10) Acceptability of a future research scan was high for parents (7–10 [10]; 88% 10/10) but lower for adolescents (5–10 [10]; 67% rated 10/10) and did not differ from acceptability for future clinical scans Post scan discomfort questionnaire (n = 18 adolescents): No discomfort (8) Mild discomfort (6) Moderate discomfort (2) High discomfort (2). One adolescent with 9/10 discomfort due to noise also reported the highest worry (6/10) and lowest acceptability of future research scans (5/10) No relationship between pain intensity immediately before scanning and discomfort (Spearman’s r = 0.13, *p* = 0.7; n = 12) or between previously completed PI-ED scores and worry during MRI (r = 0.33, *p* = 0.18; n = 18) Ease of understanding of instructions (18 adolescents) Easy to understand (15) Difficult to understand (3). Two adolescents reporting difficulty understanding instructions (0/10) also had lower ratings for future scan acceptability (5–7/10)	Parents: High acceptability of the scan (NRSs: 10–10) High willingness to undergo another MRI (NRSs: 7–10)
8. Everts, 2020 [35]	Fear: None: 55.2% Almost none: 26.9% A little: 17.5% Considerable: 0.5% High: 0.0% Discomfort: None: 32.5% Almost none: 41.4% A little: 22.6% Considerable: 3.3% High: 0.0% Discomfort significantly different for sex variable (higher in males, *p* = 0.036), but not for fear (*p* = 0.703). For controls no sex difference for discomfort (*p* = 0.714) nor fear (*p* = 0.631). In controls, fear and discomfort were positively associated. In patients, age was negatively associated with discomfort whereas maternal education correlated positively with cognitive self-control and paternal education was positively associated with IQ Longitudinal analyses: Development of fear and discomfort between childhood and adolescence showed that fear during MRI decreased significantly between childhood and adolescence in patients (*p* = 0.018), but not in controls (*p* = 0.222) Perceived discomfort remained stable between childhood and adolescence	No difference between parents and controls in perceived fear or discomfort In patients, maternal education correlated positively with cognitive self-control (inhibition) and paternal education was positively associated with IQ
9. Heathcote 2021 [25]	At baseline, CRIES-8 score was high (10.5/18), and girls reported more severe scanxiety than boys (*p* = 0.04). Higher scanxiety was associated with higher daily fear of cancer recurrence (r = 0.522) and stress (r = 0.435). Higher baseline fear of cancer recurrence was linked to higher daily fear of cancer recurrence (r = 0.734), higher perceived stress (r =0.355), higher bodily threat monitoring (r = 0.361), higher self-checking behaviors (r = 0.327), higher social interaction (r = 0.373) and lower positive affect (r = 0.379).	NR
10. Webster, 2025 [13]	Mean FCR on scan day was significantly higher than FCR in the pre-scan phase, (*p* = 0.01) (Mbase phase). Furthermore, there was a significant increase in FCR in the days leading up to scan day, *p* = 0.01 (Mbase day). Mean FCR in the post-scan phase was significantly lower than mean FCR on scan day, *p* = 0.01 (Mbase phase) and significantly lower than mean FCR in the pre-scan phase, *p* = 0.00 (Mbase phase). Furthermore, there was a significant decrease in FCR in the days following scan day, *p* = 0.01 (Mbase day). The positive relationship observed between bracing and FCR on scan day was preserved during the pre-scan phase, as indicated by a nonsignificant difference in this relationship at the pre-scan phase compared to scan day, *p* = 0.25 (Mbracing phase). There was a significant relationship between bracing and FCR slope (*p* = 0.01) (Mbracing day). The positive relationship observed between bracing and FCR on scan day was different in the post-scan phase, as indicated by a significant difference in this relationship at the post-scan phase compared to scan day, (*p* = 0.01) (Mbracing phase). There was a significant relationship between bracing and FCR slope, indicating that those who braced more showed a sharper decrease in FCR in the days following the scan compared to those who braced less (*p* = 0.01) (Mbracing day). There was no significant relationship between positive uncertainty management (hope and optimism) and FCR on scan day, (*p* = 0.44) (MposExpMan phase). The null relationship between positive uncertainty management and FCR on scan day was also present at the pre-scan phase, (*p* = 0.46) (MposExpMan phase), indicating that positive uncertainty management was not related to FCR levels in the pre-scan phase. Furthermore, FCR slope in the days leading up to scan day was not significantly related to positive uncertainty management, (*p* = 0.85) (MposExpMan day). Null effects were present at the post-scan phase, (*p* = 0.79) (MposExpMan phase), indicating that positive uncertainty management was not related to FCR levels in the post-scan phase. Furthermore, FCR slope in the days following the scan was not significantly related to positive uncertainty management, (*p* = 0.49) (MposExpMan day)	NR

Note: NR = Not reported; SDS = Standard Deviation Score; FCR = Fear of cancer recurrence.

## Data Availability

No new data were created or analyzed in this study.

## Supplementary Materials

The following supporting information can be downloaded at: https://www.mdpi.com/article/10.3390/healthcare14091160/s1, Supplementary Material S1. This document presents the search strategy used in this review. Supplementary Material S2. This document presents the reason of each risk of bias score by studies. Supplementary Material S3. PRISMA checklist.
