# Supplementary material for: Biomarkers and Psychological Factors Associated with Distress in Children, Adolescents, and Young Adults Undergoing MRI Neuroimaging: A Systematic Review of Observational Studies with Clinical Recommendations
Source: Healthcare (Basel). 2026 Apr 25;14(9):1160. doi: 10.3390/healthcare14091160 (PMC13163460; doi:10.3390/healthcare14091160)
Supplement: Supplementary file 1 [file healthcare-14-01160-s001.zip › Suppementary Material S2. QUIPS with reason-R1.pdf]

Supplementary Material S2. Risk of Bias analysis by Quality Prognostic Studies Tool (QUIPS) with reason

	Study	Dominios											
		**1. Study Participation**		**2. Study Attrition**		**3. Prognostic Factor Measurement**		**4. Outcome Measurement**		**5. Study Confounding**		**6. Statistical Analysis and Reporting**	
		Judgement	Reason	Judgement	Reason	Judgement	Reason	Judgement	Reason	Judgement	Reason	Judgement	Reason
1	Westra, 2011 [20]	Low	There are a good participants description, recruitment acording eligility criteria. The study sample represent adequatly the population of interest without potential risk of bias	Hight	There are not explanations for dropouts or reasons of dropouts. Authors did not complete an analysis comparing completers and non-completers.	Low	There are a clear definition of PF, mesurement is clear and it perform the same way to all sample.	Low	The outcome is meausure in the same way to all participants. It provides a clear outcome definition and valid-reliable instrument with the same method - setting measurement in all study participants.	Moderate	It the study it was included the age as confounding factors but not other potential factors as procedure time, type of analgesia… The method and procedure to analys the factor age, it was correctly.	Moderate	The statistical analysis is adequate for all primary outcome, but it was interesant include a multivariante analysis to explore cofounding factors interaction.

2	Haddad, 2013 [14]	Low	There are a good participants description, recruitment acording eligility criteria. The study sample represent adequatly the population of interest without potential risk of bias	High t	There are not explanations for dropouts or reasons of dropouts. Authors did not complete an analysis comparing completers and non-completers.	Low	There are a clear definition of PF, mesuremen t is clear and it perform the same way to all sample.	Mod erat e	The outcome is meausure in the same way to all participants. It provides a clear outcome definition but they uses a no validated instrument to assess outcome. The used a on-line questionaire that could be introduce a remomoring bias in the self-report. The used the same method - setting measuremen t in all study participants.	High t	It was included the nervousness as confounding factors but not other potential factors as age, procedure time, type of analgesia… The method and procedure to analys the factor nervousness, it was correctly but they don´t uses a valid-reliable scale, that makes it difficult to compare with other evaluations.	Mod erat e	The statistical analysis is adequate for all primary outcome, but it was interesant include a multivariante analysis to explore cofounding factors interaction.

3	Chou, 2014 [5]	Low	There are a good participants description, recruitment acording eligility criteria. The study sample represent adequatly the population of interest without potential risk of bias	Moderate	There provide number of dropouts but not reasons. Authors did not complete an analysis comparing completers and non-completers.	Low	The analyze potential diference in age o sex, in all sample.	Moderate	The outcome is meausure in the same way to all participants. It provides a clear outcome definition but they uses a no validated specific instrument to assess outcome. The used a on-line questionaire that could be introduce a remomoring bias in the self-report. The used the same method - setting measurement in all study participants.	Hight	They don´t analyze potential cofounding factors that can influce in direction or size of relation into prognostic factor and outcome	Moderate	The statistical analysis is adequate for all primary outcome, but it was interesant include a multivariante analysis to explore cofounding factors interaction.

4	Staphorst, 2015 [7]	Low	There are a good participants description, recruitment acording eligility criteria. The authors provide information about participants´ previous studies included. The study sample represent adequatly the population of interest without potential risk of bias	Low	There provide number of dropouts and reasons. Authors did not complete an analysis comparing completers and non-completers, but there are only two non-completers that its dificult it can introduce a bias in the conclusion´s analysis	ND	ND	Low	The outcome is meausure in the same way to all participants (semi-structured interview).	Low	The analyze potential diference in age and medical condition, in all sample.	ND	ND
5	Staphorst, 2017 [6]	Low	There are a good participants description, recruitment acording eligility criteria. The authors provide information about participants clinical condition. The	Low	There provide number of dropouts and reasons. Authors did not complete an analysis comparing completers and non-completers, but there are only two non-completers that	Low	There are a clear definition of traix anxiety and child behavior, mesurement is clear and it perform the same way to all sample.	Low	The outcome is meausure in the same way to all participants. It provides a clear outcome definition and valid-reliable instrument (Discomfort during research	Low	The analyze potential diference in age and medical condition, in all sample.	Moderate	The statistical analysis is adequate for all primary outcome, but it was interesant include a multivariante analysis to explore cofounding factors interaction.

			study sample represent adequatly the population of interest without potential risk of bias		its dificult it can introduce a bias in the conclusion´s analysis				procedures CDRPQ) with the same method - setting measurement in all study participants.				
6	Jaite, 2019 [33]	Low	There are a good participants description, recruitment acording eligility criteria. The authors provide information about participants cutoff clinical condition. The study sample represent adequatly the population of interest without potential risk of bias	Hight	There are not explanations for dropouts or reasons of dropouts. Authors did not complete an analysis comparing completers and non-completers.	Low	There are a clear definition of anxiety disorder and physiological arousal, mesurement is clear and it perform the same way to all sample.	Low	The outcome is meausure in the same way to all participants. It provides a clear outcome definition and valid-reliable instruments psichological (Patient Experience Questionnaire PEQ) and objective (Heart Rate, Blood pressure) with the same method - setting measuremen	Low	The analyze potential diference in age, sex, and IQ, in all sample.	Low	The statistical analysis is adequate for all primary outcome, and it analyze cofactors interaction (sex, age, IQ)

									t in all study participants.				
7	Verriotis, 2020 [34]	Low	There are a good participants description, recruitment acording eligility criteria. The authors provide information about participants cutoff clinical condition. The study sample represent adequatly the population of interest without potential risk of bias	Low	There provide number of dropouts and reasons. Authors did complete an analysis comparing completers and non-completers.	Low	There are a clear definition of pain intesity, worry quality of life and catastrophizing. The mesurement is clear and it perform the same way to all sample.	Low	The outcome is meausure in the same way to all participants. It provides a clear outcome definition and valid-reliable instruments of distress	Low	The analyze potential diference in age, sex, and IQ, in all sample.	Low	The statistical analysis is adequate for all primary outcome, and it analyze cofactors interaction (sex, age, pain duration)

8	Everts, 2020 [35]	Moderate	There are a good participants description, recruitment acording eligility criteria. The authors provide information about participants clinical condition. The group of "patients" represent a very specific pathology population, which can have cognitive alteration or more procedimental fear due to thier clinical condition versus other more prevalent pathologies. This "patient group" could introduce a selection bias and its dificult	Hight	There are not explanations for dropouts or reasons of dropouts. Authors did not complete an analysis comparing completers and non-completers.	Low	There are a clear definition of parental education, cognitive self-control. The mesurement is clear and it perform the same way to all sample.	Low	The outcome is meausure in the same way to all participants. It provides a clear outcome definition and valid-reliable instruments of distress (5 points scale)	Low	The analyze potential diference in age, sex, clinical condition, in all sample.	Moderate	The statistical analysis is adequate for all primary and secondary outcome, but it was interesant include a multivariante analysis to explore cofounding factors interaction.

to share their result to other more prevalente childhood pathologies.
