# Supplementary material for: Biomarkers and Psychological Factors Associated with Distress in Children, Adolescents, and Young Adults Undergoing MRI Neuroimaging: A Systematic Review of Observational Studies with Clinical Recommendations
Source: Healthcare (Basel). 2026 Apr 25;14(9):1160. doi: 10.3390/healthcare14091160 (PMC13163460; doi:10.3390/healthcare14091160)
Supplement: Supplementary file 1 [file healthcare-14-01160-s001.zip › Supplementary Material S1. Search strategy.pdf]

Search strategy

Medline

Brain image capture/ Intervention

1. (“Neuroimaging” OR “Neuroimage” OR “Image brain”)
2. (“Neuroimag* OR “Brain Imag*”)
3. (“MRI” OR “fMRI” OR “MRI scan” OR “Resonance Magnetic” OR “Magnectic Resonance imaging”)
4. (“Scan” OR “Scan process” OR “brain scan” OR “Brain scanner”)

Population/Sample

5. (pediatric* or paediatric* or child* or teen* or preteen* or adolescen* or youth* or youngster* or young person* or young people or juvenile*)
6. Limit to “child (4-18 years)”

Type of study

7. (“Case-control” OR “Observational” OR “Cross-sectional”)
8. (“Prospective study”)
9. Limit to English language
10. Limit to yr “2010-current”

Outcomes

11. (“Factors affecting scanner”)
12. (“Distress factors”)
13. (“Psychosocial factors”)
14. (“Motion head”)
15. (“Problems during scanner process”)
16. (“Rated discomford” OR “Perceived Risk” OR “Acceptability”)

Web of science

Intervention: Brain image capture

#1 TS/TI/AB= (Neuroimaging OR Neuroimage OR Image brain)

#2 TS/TI/AB = (Neuroimag* OR Brain Imag*)

#3 TS/TI/AB = (“MRI” OR “fMRI” OR “MRI scan” OR “Resonance Magnetic” OR “Magnectic Resonance imaging”)

#4 TS/TI/AB = (Scan OR Scan process OR brain scan OR Brain scanner)

Population/Sample

#5 TS/TI/AB= (pediatric* or paediatric* or child* or teen* or preteen* or adolescen* or youth* or youngster* or young person* or young people or juvenile*)

#6 TS/TI/AB= (4-18 years)

Type of study

#11 (Case-control OR Observational OR Cross-sectional)

#12(Prospective study”)

#13 Limit to14 to English language

#14 Limit to 20 to yr “2010-current”

Outcomes

#15 TS= (Factors affecting scanner process)

#16 TS= (Problems during scanner process)

#17 TS= (Distress factors)

#18 TS= (Psychosocial factors)

#19 TS= (Motion head)

#20 TS= (Rated discomfort OR Perceived Risk OR Acceptability)

Cochrane Library

Intervention: Brain image capture

Neuroimaging [Mesh Term] -All tree

Functional Neuroimaging [Mesh Term] -All tree

Brain Mapping [Mesh Term] -All tree

Magnetic Resonance Imaging [Mesh Term] -All tree

Diffusion Magnetic Resonance Imaging [Mesh Term] -All tree

Population/Sample

Child [Mesh Term] -All tree

Pediatrics [Mesh Term] -All tree

Psychology, Child [Mesh Term] -All tree

Psychology, Adolescent [Mesh Term] -All tree

Outcomes

Psychological Distress [Mesh Term] -All tree

Fear [Mesh Term] -All tree

Anxiety [Mesh Term] -All tree

Anxiety Disorders [Mesh Term] -All tree

Mood Disorders [Mesh Term] -All tree
